# Finding potential lncRNA–disease associations using a boosting-based ensemble learning model

**DOI:** 10.3389/fgene.2024.1356205

**Published:** 2024-03-01

**Authors:** Liqian Zhou, Xinhuai Peng, Lijun Zeng, Lihong Peng

**Affiliations:** ^1^ School of Computer Science, Hunan University of Technology, Zhuzhou, Hunan, China; ^2^ School of Computer Science, Hunan Institute of Technology, Hengyang, China

**Keywords:** lncRNA–disease association, singular value decomposition, LightGBM, AdaBoost, convolutional neural network

## Abstract

**Introduction:** Long non-coding RNAs (lncRNAs) have been in the clinical use as potential prognostic biomarkers of various types of cancer. Identifying associations between lncRNAs and diseases helps capture the potential biomarkers and design efficient therapeutic options for diseases. Wet experiments for identifying these associations are costly and laborious.

**Methods:** We developed LDA-SABC, a novel boosting-based framework for lncRNA–disease association (LDA) prediction. LDA-SABC extracts LDA features based on singular value decomposition (SVD) and classifies lncRNA–disease pairs (LDPs) by incorporating LightGBM and AdaBoost into the convolutional neural network.

**Results:** The LDA-SABC performance was evaluated under five-fold cross validations (CVs) on lncRNAs, diseases, and LDPs. It obviously outperformed four other classical LDA inference methods (SDLDA, LDNFSGB, LDASR, and IPCAF) through precision, recall, accuracy, F1 score, AUC, and AUPR. Based on the accurate LDA prediction performance of LDA-SABC, we used it to find potential lncRNA biomarkers for lung cancer. The results elucidated that 7SK and HULC could have a relationship with non-small-cell lung cancer (NSCLC) and lung adenocarcinoma (LUAD), respectively.

**Conclusion:** We hope that our proposed LDA-SABC method can help improve the LDA identification.

## 1 Introduction

Long non-coding RNAs (lncRNAs) are important RNA molecules comprising more than 200 nucleotides (Jiang et al., 2015; Liu et al., 2021; Chen et al., 2023). lncRNAs have been in the clinical use as prognostic biomarkers of many complex diseases, including cancers (Tang et al., 2022; 2021; Huo et al., 2021). For example, liver-specific lncRNA FAM99A plays a cancer-inhibiting role in hepatocellular carcinoma and might serve as its prognostic biomarker (Mo et al., 2022). Exosomal RP5-977B1 might be a diagnostic biomarker of non-small-cell lung cancer (NSCLC) (Min et al., 2022). MALAT1 has been broadly applied for its oncogenic properties in lung cancer (Xin et al., 2023), bladder cancer (Li et al., 2017), breast cancer (Adewunmi et al., 2023), and ovarian cancer (Mao et al., 2021). Identifying possible relationships between lncRNAs and diseases helps capture potential biomarkers for various cancers and provide clues for their diagnosis and treatment (Wang et al., 2021). Traditional wet experiments for detecting new lncRNA–disease associations (LDAs) are costly and have low success rates; computational techniques have been increasingly developed to discover new LDAs (Chen et al., 2021; Zhao et al., 2023). Meanwhile, various lncRNA-related databases, such as MNDR v2.0 (Cui et al., 2018), Lnc2Cancer (Ning et al., 2016), LncRNADisease 3.0 (Lin et al., 2023b), and NRED (Dinger et al., 2009), provide diverse LDA data resources. Based on these resources, many computational methods, especially network-based and machine learning methods, have been applied to LDA prediction (Chen et al., 2017; Chen and Huang, 2022; Sheng et al., 2023).

Network-based methods predict new LDAs through label propagation and multi-information fusion on the heterogeneous lncRNA–disease networks (Jiang et al., 2010; Zou et al., 2016; Hu et al., 2017; Wang et al., 2019; Yu et al., 2020; Qiu et al., 2023b). Chen et al. conducted many research studies and significantly promoted LDA prediction (Chen and Yan, 2013; Chen et al., 2015; Chen, 2015a; Chen, 2015b). Based on these studies, they comprehensively concluded the current computational methods for non-coding RNA analysis and unfolded existing challenges and corresponding solutions (Chen and Huang, 2022; 2023). Xie et al. used the unbalanced bi-random walk algorithm (Xie et al., 2020b; a) and bidirectional linear neighborhood label propagation (Xie et al., 2023) for LDA identification. In addition, a random walk with a restart algorithm (Wang et al., 2022) has been still applied to find new LDAs. Network-based methods found many possible LDAs, but they did not analyze the topological features of LDA networks.

Machine learning methods have been applied to various association discovery tasks (Zou et al., 2018; Peng et al., 2019; 2022b; Shen et al., 2022; Wu et al., 2022; Yu et al., 2022; Lin et al., 2023a; Peng et al., 2023a; Peng et al., 2023b; Peng et al., 2024b; Han et al., 2023; Liu and Zhang, 2023; Qi and Zou, 2023; Xiong et al., 2023; Xu et al., 2024; Zhang et al., 2024). Consequently, machine learning algorithms have been broadly applied in LDA prediction, for example, collaborative filtering (Yu et al., 2019), graph regularization (Liu et al., 2020; Wang et al., 2021), matrix factorization (Fu et al., 2018; Wang et al., 2020; Xi et al., 2022), heterogeneous graph learning framework, (Cao et al., 2023), and ensemble learning models (Peng et al., 2022a). Notably, deep learning has been broadly applied due to its powerful classification performance (Sun et al., 2022; Wang et al., 2023; Wang et al., 2023b; Hu et al., 2023; Jiang et al., 2023; Zhang et al., 2023; Zhang and Wu, 2023; Zhou et al., 2024a), such as in the graph convolution network (Wang W. et al., 2022), node2vec (Li et al., 2021), collaborative deep learning (Lan et al., 2020), deep neural network (Wei et al., 2020), deep multi-network embedding (Ma, 2022), graph autoencoder (Liang et al., 2023; Zhou et al., 2024b), and a capsule network with the attention mechanism (Zhang et al., 2023). In particular, to identify new LDAs, a few models first extracted LDA features and classified unknown lncRNA–disease pairs (LDPs) by combining machine leaning models. SDLDA (Zeng et al., 2020) effectively integrated deep learning and singular value decomposition (SVD), LDASR (Guo et al., 2019) combined autoencoder and rotating forest, LDNFSGB (Zhang et al., 2020) used autoencoder and the gradient boosting model, IPCARF (Zhu et al., 2021) applied the incremental principal component analysis and random forest, CapsNet-LDA (Zhang et al., 2023) utilized stacked autoencoder and attention mechanism, and LDAEXC (Lu and Xie, 2023) integrated deep autoencoder and XGBoost. Machine learning-based methods boosted LDA prediction, but they neglect noisy and irrelevant data.

To boost the LDA prediction performance, here, we developed LDA-SABC, a novel boosting-based framework for LDA prediction. LDA-SABC extracts LDA features based on SVD and classifies LDPs by integrating LightGBM (Wang et al., 2023) and AdaBoost combined with the convolutional neural network (AdaBoost-CNN) (Taherkhani et al., 2020; Peng et al., 2023c). The LDA-SABC performance was evaluated under fivefold cross validations (CVs) on lncRNAs, diseases, and LDPs. This approach accurately found a few potential lncRNAs for lung cancer. LDA-SABC is publicly available at https://github.com/plhhnu/LDA-SABC.

## 2 Materials and methods

### 2.1 Overview of LDA-SABC

**FIGURE 1:**
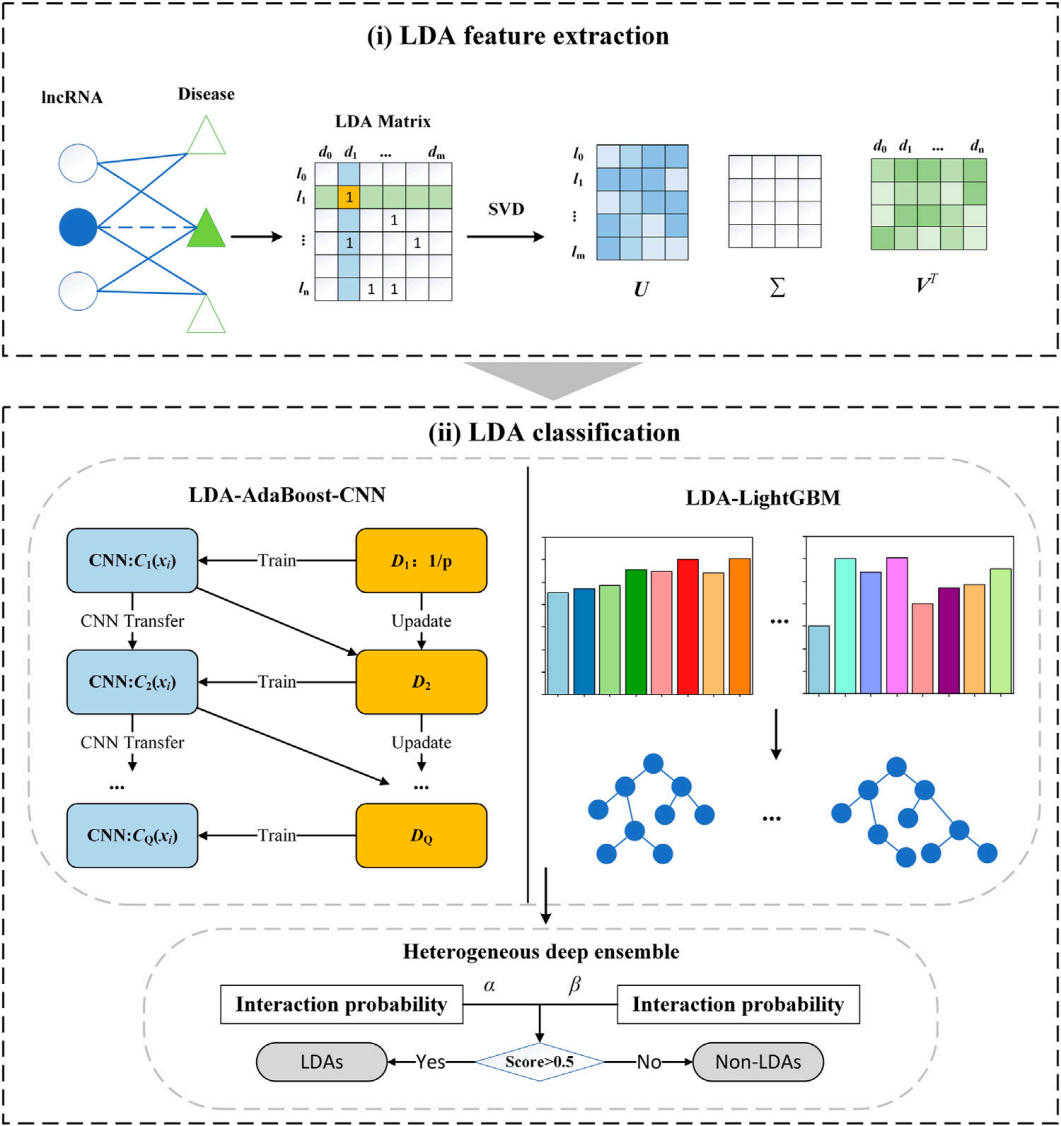
Flowchart of the LDA prediction model LDA-SABC: (i) LDA feature extraction. (ii) LDA classification.

LDA-SABC contains two main steps: 1) LDA feature extraction: the LDP linear features are extracted through SVD. 2) LDA classification: the association probability of each LDP is computed by integrating AdaBoost-CNN and LightGBM. The details are shown in Figure 1.

### 2.2 Data preparation

LDA-SABC was evaluated on two human LDA datasets (Peng et al., 2024a), namely, LncRNADisease (Chen et al., 2012) and MNDR (Cui et al., 2018). After deleting diseases without regular names or MeSH data and lncRNAs without sequence data, the number of lncRNAs, one of the diseases, and one of the LDAs in two LDA datasets are listed in Table 1. Subsequently, an LDA network containing *n* lncRNAs and *m* diseases is denoted as $Y\in R^{n\times m}$ , where *y*_*ij*_ = 1 if lncRNA *l*_*i*_ is associated with disease *d*_*j*_, otherwise *y*_*ij*_ = 0.

**TABLE 1:** Introduction of two LDA datasets.

Dataset	lncRNA	Disease	LDA
LncRNADisease	82	157	605
MNDR	89	190	1,529

### 2.3 LDA feature extraction

SVD (Abdi, 2007) can effectively extract features by eigen decomposition. By selecting larger singular values, SVD can reduce the dimensionality of the data and remove features that contribute less to data variability, thereby reducing the storage and calculation costs of the data. In addition, the feature vectors corresponding to smaller singular values represent noise or redundant parts in the data. By selecting larger singular values, SVD can retain the main linear features, thereby removing noise and redundant information. Furthermore, the size of singular values represents important features in the data, and SVD helps us understand the structure and variation patterns of the data by observing the size of singular values and their corresponding feature vectors. Thus, SVD is used to extract lncRNA and disease features: the LDA matrix $Y\in R^{n\times m}$ is factorized using Eq. 1:

$$Y=U\sum V^{T},$$ (1)

where ***V*** ^*T*^ represents the transpose of ***V***, ***U*** ∈ *R* ^*n*×*n*^ and ***V*** ∈ *R* ^*m*×*m*^ are two real matrices, and Σ denotes a diagonal matrix composed of *n* singular values.

Subsequently, the *e* largest singular values are selected to build an approximation representation using Eq. 2:

$$R\approx U_{i}\sum_{e}\;{\left(V_{j}\right)}^{T}.$$ (2)

Consequently, ***U***_*i*_ and ${V_{j}}^{T}$ denote the features of the *i*th lncRNA *l*_*i*_ and the *j*th disease *d*_*j*_, respectively.

As a result, the features of each lncRNA can be represented as an *a*-dimensional vector, and the features of each disease can be represented as a *b*-dimensional vector. The two features are concatenated as a *d* (*d* = *a* + *b*)-dimensional vector for characterizing each LDP.

### 2.4 LDA prediction

For an LDA dataset $D=\left(X,\hat{Y}\right)$ , with *p* (*p* = *n* × *m*) samples (i.e., *p* LDPs), let ***x***_*i*_ ∈ ***X*** denote the *i*th LDP with *d*-dimensional features, and $y_{i}\in \hat{Y}$ denotes its label.

#### 2.4.1 LDA-AdaBoost-CNN

Inspired by AdaBoost-CNN proposed by Hastie et al. (2009) and Taherkhani et al. (2020), we exploit an LDA identification algorithm LDA-AdaBoost-CNN by integrating AdaBoost and CNNs based on transfer learning. Given *Q* CNNs, LDA-AdaBoost-CNN uses CNNs as base estimators for predicting LDAs. During training, we use a vector ***D*** with initial values $\frac{1}{p}$ to measure the importance of each sample. Next, the weights of all training samples are updated and normalized. Finally, LDA-AdaBoost-CNN outputs a binary vector $o_{l}^{k}\left(x_{i}\right)$ with the last CNN to identify one LDP as LDA (*k* = 1) or non-LDA (*k* = 2).

For the *i*th feature map in the *l*th layer $y_{i}^{l}$ , its activity is computed using Eq. 3:

$$y_{i}^{l}=\sum_{j}f\left(w_{i,j}^{l}*y_{j}^{l-1}+b_{i}^{l}\right),$$ (3)

where $w_{i,j}^{l}$ represents the weight of a convolutional kernel, which maps the *j*th feature at the $\left(l-1\right)$ th CNN layer to the *i*th feature at the *l*th CNN layer, and $b_{i}^{l}$ is the bias of the *i*th feature in the *l*th layer. Finally, the output ***F*** ^*l*^ at the *l*th hidden layer is computed using Eq. 4:

$$F^{l}=f\left(W^{l}{\left(F^{l-1}\right)}^{T}+b^{l}\right),$$ (4)

where $f\left(\cdot \right)$ denotes a non-linear function. Consequently, the probability distribution matrix ***Z*** of all LDPs is computed via a softmax function using Eq. 5:

$$Z=softmax\left(W^{o}{\left(F^{L}\right)}^{T}+b^{o}\right),$$ (5)

where ***W*** ^*o*^ denotes a weight matrix linking the last hidden layer with the output layer, ***b*** ^*o*^ indicates the bias, and ***F*** ^*L*^ represents the output at the last hidden layer.

For the *i*th sample ***x***_*i*_, after training *Q* CNNs, its output is computed based on its output $o_{q}^{k}\left(x_{i}\right)\;\left(k=1,2\right)$ in the *q*th CNN using Eq. 6:

$$C\left(x_{i}\right)=\underset{k}{argmax}\sum_{q=1}^{Q}c_{k}^{q}\left(x_{i}\right),$$ (6)

where

$$c_{k}^{q}\left(x_{i}\right)=\log\left(o_{k}^{q}\left(x_{i}\right)\right)-\frac{1}{2}\sum_{k^{\prime }=1}^{2}\log\left(o_{k^{\prime }}^{q}\left(x_{i}\right)\right).$$ (7)

#### 2.4.2 LDA-LightGBM

LightGBM is a gradient-based model. It uses two powerful techniques to acquire the optimal split node and accurately classify unknown samples: one-side sampling and exclusive feature bundling. Here, inspired by LightGBM (Ke et al., 2017), we propose a LightGBM-based LDA inference algorithm LDA-LightGBM. First, gradients of all LDPs in the training set are computed, and the *a%* LDPs with the smallest gradients are taken as *A*. Next, a sample set *B* is constructed by randomly selecting *b* ×|*A* ^*r*^| samples from the remaining LDPs *A* ^*r*^. Finally, all LDPs are split on the node *p*_*d*_ according to information gain *I*_*j*_ (*p*_*d*_) on *A* ∪ *B* using Eq. 8:

$$I_{j}\left(p_{d}\right)=\frac{1}{p}\left(\frac{{\left(\sum_{x_{i}\in A_{l}}g_{i}+\frac{1-a}{b}\sum_{x_{i}\in B_{l}}g_{i}\right)}^{2}}{p_{r}^{j}\left(d\right)}\right)+\frac{1}{p}\left(\frac{{\left(\sum_{x_{i}\in A_{r}}g_{i}+\frac{1-a}{b}\sum_{x_{i}\in B_{r}}g_{i}\right)}^{2}}{n_{r}^{j}\left(d\right)}\right),$$ (8)

where *A*_*l*_ = {*x*_*i*_ ∈ *A*: *x*_*ij*_ ≤ *p*_*d*_}, *A*_*r*_ = {*x*_*i*_ ∈ *A*: *x*_*ij*_ > *p*_*d*_}, *B*_*l*_ = {*x*_*i*_ ∈ *B*: *x*_*ij*_ ≤ *p*_*d*_}, *B*_*r*_ = {*x*_*i*_ ∈ *B*: *x*_*ij*_ > *p*_*d*_}, and *g*_*i*_ represents the negative gradient.

However, LDA features have high dimensions and multiple zero values, that is, the features cannot simultaneously have nonzero values. To solve this problem, LRI-LightGBM first uses weights to characterize the whole conflict between all LDA features and construct a weighted graph. Subsequently, all LDA features are sorted and are set to a defined bundle or create a new bundle. Finally, all LDPs are classified using Eq. 9:

$$F_{I_{q}}\left(x_{i}\right)=\sum_{q=1}^{I_{q}}\gamma_{q}h_{q}\left(x_{i}\right),$$ (9)

where *T*_*q*_ is the maximum iteration number and $h_{q}\left(x_{i}\right)$ is the *q*th basic decision tree.

#### 2.4.3 Ensemble learning

Ensemble learning exhibits strong classification performance compared to individual classifiers. Thus, we combined LDA-AdaBoost-CNN and LDA-LightGBM for LDA identification. For one LDP ***x***_*i*_, let *C*(***x***_*i*_) and *F*(***x***_*i*_) represent its association scores computed by LDA-AdaBoost-CNN and LDA-LightGBM, respectively; its final association probability *p*(***x***_*i*_) is obtained Eq. 10:

$$P\left(x_{i}\right)=\alpha C\left(x_{i}\right)+\beta F\left(x_{i}\right),$$ (10)

where *α* and *β*(*β* = 1 − *α*) are used to evaluate the importance of LDA-AdaBoost-CNN and LDA-LightGBM with respect to the LDA inference performance, respectively.

## 3 Results

### 3.1 Experimental settings

To assess the LDA inference performance of LDA-SABC, we implemented three fivefold CVs to compare it with four representative LDA prediction approaches, namely, SDLDA (Zeng et al., 2020), LDNFSGB (Zhang et al., 2020), IPCARF (Zhu et al., 2021), and LDASR (Guo et al., 2019). The parameters in the above four methods were derived from their corresponding literatures. For the LDA-SABC model, we set n_estimators, learning rate, and epochs to 100, 0.1, and 10, respectively, in LDA-AdaBoost-CNN and n_estimators and learning rate to 100 and 0.1, respectively, in LRI-LightGBM. The dimension *d* of an LDA feature vector was set to 64.

### 3.2 Comparison with four classical LDA prediction methods

**FIGURE 2:**
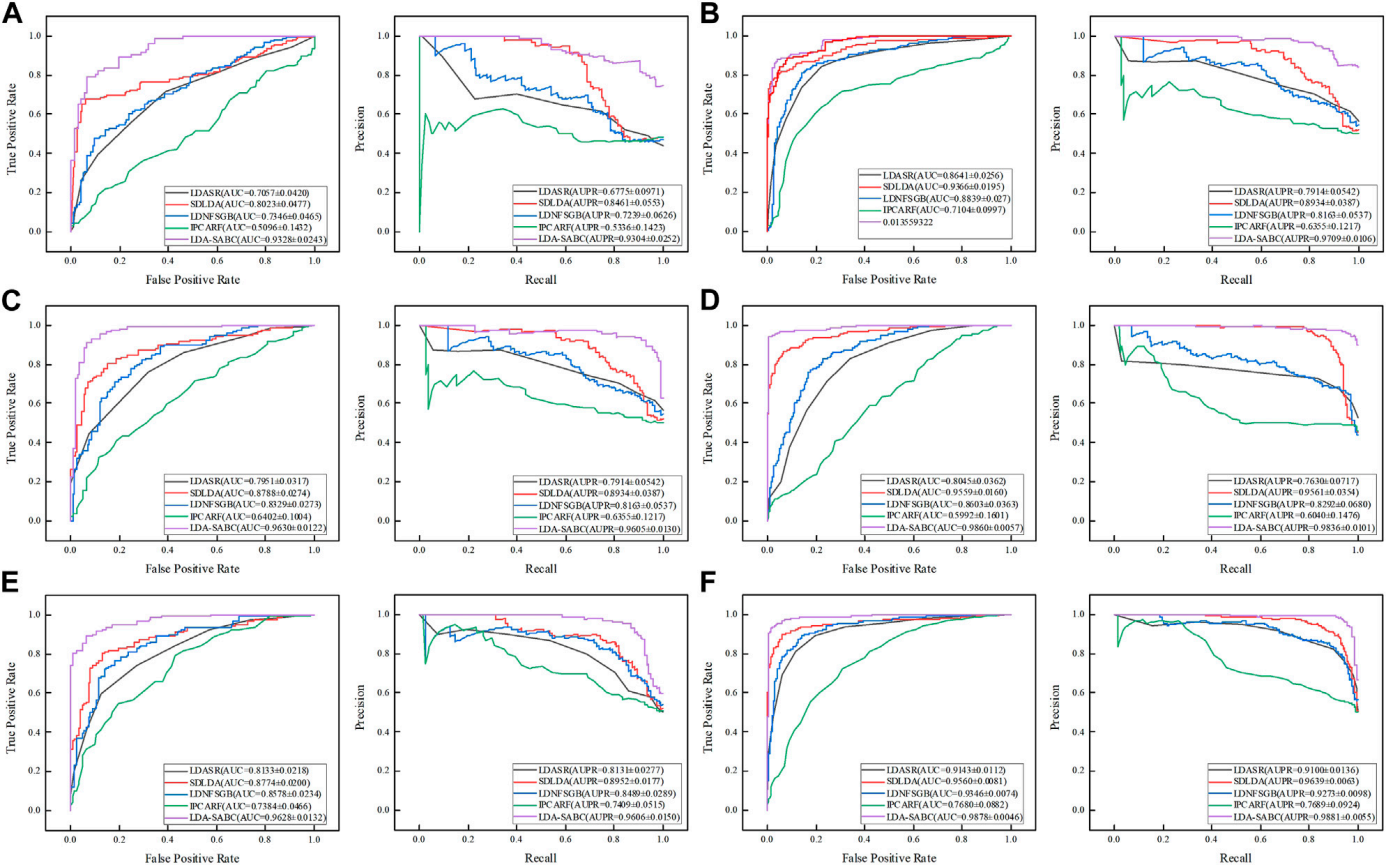
ROC and PR curves of LDA-SABC and four other methods: **(A,B)** ROC and PR curves on the LncRNADisease and MNDR databases under *CV*_*l*_, respectively. **(C,D)** Curves under *CV*_*d*_. **(E,F)** Curves under *CV*_*ld*_.

We used six evaluation metrics (precision, recall, accuracy, F1 score, AUC, and AUPR (Shen et al., 2022; Liu et al., 2023; Qiu et al., 2023a)) to assess the performance of LDA-SABC and four other LDA prediction algorithms (SDLDA, LDNFSGB, IPCARF, and LDASR) under three different fivefold cross validations. The three CVs are fivefold CV on lncRNAs (*CV*_*l*_), five-fold CV on diseases (*CV*_*d*_), and fivefold CV on LDPs (*CV*_*ld*_). The details refer to Peng et al. (2024a). Tables 2–4 depict the performance of LDA-SABC and four other methods on two databases (i.e., LncRNADisease and MNDR) under the three CVs. Figure 2 characterizes the corresponding ROC and precision–recall (PR) curves.

**TABLE 2:** Performance of five LDA inference methods under CV_l_.

	Dataset	SDLDA	LDNFSGB	IPCARF	LDASR	LDA-SABC
Precision	LncRNADisease	0.8514 ± 0.0509	0.7004 ± 0.0639	0.4878 ± 0.1309	0.6726 ± 0.1200	**0.8980 ± 0.0306**
	MNDR	0.9399 ± 0.0154	0.8552 ± 0.0393	0.6615 ± 0.0966	0.8405 ± 0.0300	**0.9494 ± 0.0172**
Recall	LncRNADisease	0.6521 ± 0.0732	0.6092 ± 0.0790	0.5721 ± 0.1580	0.5129 ± 0.0946	**0.7709 ± 0.0622**
	MNDR	0.8239 ± 0.0437	0.8021 ± 0.0498	0.6434 ± 0.1545	0.7358 ± 0.0562	**0.8436 ± 0.0513**
Accuracy	LncRNADisease	0.7799 ± 0.0341	0.6769 ± 0.0423	0.4906 ± 0.0951	0.6417 ± 0.0597	**0.8444 ± 0.0445**
	MNDR	0.8857 ± 0.0283	0.8323 ± 0.0230	0.6526 ± 0.0775	0.7972 ± 0.0268	**0.8989 ± 0.0317**
F1 score	LncRNADisease	0.7365 ± 0.0563	0.6462 ± 0.0451	0.5125 ± 0.1100	0.5668 ± 0.0536	**0.8278 ± 0.0363**
	MNDR	0.8775 ± 0.0278	0.8260 ± 0.0230	0.6401 ± 0.1017	0.7827 ± 0.0260	**0.8925 ± 0.0307**
AUC	LncRNADisease	0.8023 ± 0.0477	0.7346 ± 0.0465	0.5096 ± 0.1432	0.7057 ± 0.0420	**0.9328 ± 0.0243**
	MNDR	0.9366 ± 0.0195	0.8839 ± 0.0270	0.7104 ± 0.0997	0.8641 ± 0.0256	**0.9675 ± 0.0147**
AUPR	LncRNADisease	0.8461 ± 0.0553	0.7239 ± 0.0626	0.5336 ± 0.1423	0.6775 ± 0.0971	**0.9304 ± 0.0252**
	MNDR	0.9533 ± 0.0129	0.8832 ± 0.0307	0.7128 ± 0.1012	0.8671 ± 0.0252	**0.9709 ± 0.0106**

**TABLE 3:** Performance of five LDA inference methods under CV_d_.

	Dataset	SDLDA	LDNFSGB	IPCARF	LDASR	LDA-SABC
Precision	LncRNADisease	0.8854 ± 0.0377	0.7548 ± 0.0639	0.5583 ± 0.0910	0.7462 ± 0.0613	**0.9218 ± 0.0242**
	MNDR	0.9232 ± 0.0331	0.8005 ± 0.0625	0.5557 ± 0.1473	0.7625 ± 0.0749	**0.9573 ± 0.0217**
Recall	LncRNADisease	0.7182 ± 0.0694	0.7309 ± 0.0646	0.7538 ± 0.1067	0.6431 ± 0.0757	**0.8745 ± 0.0353**
	MNDR	0.8579 ± 0.0655	0.6936 ± 0.0794	0.5279 ± 0.1969	0.5758 ± 0.0894	**0.9231 ± 0.0400**
Accuracy	LncRNADisease	0.8187 ± 0.0282	0.7552 ± 0.0291	0.5766 ± 0.0740	0.7165 ± 0.0339	**0.9008 ± 0.0232**
	MNDR	0.9043 ± 0.0174	0.7670 ± 0.0432	0.5593 ± 0.1159	0.7010 ± 0.0463	**0.9455 ± 0.0146**
F1 score	LncRNADisease	0.7917 ± 0.0519	0.7407 ± 0.0526	0.6339 ± 0.0715	0.6873 ± 0.0512	**0.8970 ± 0.0218**
	MNDR	0.8886 ± 0.0475	0.7402 ± 0.0577	0.5190 ± 0.1434	0.6485 ± 0.0555	**0.9394 ± 0.0260**
AUC	LncRNADisease	0.8788 ± 0.0274	0.8329 ± 0.0273	0.6402 ± 0.1004	0.7951 ± 0.0317	**0.9630 ± 0.0122**
	MNDR	0.9559 ± 0.0160	0.8603 ± 0.0363	0.5992 ± 0.1601	0.8045 ± 0.0362	**0.9860 ± 0.0057**
AUPR	LncRNADisease	0.8934 ± 0.0387	0.8163 ± 0.0537	0.6355 ± 0.1217	0.7914 ± 0.0542	**0.9605 ± 0.0130**
	MNDR	0.9561 ± 0.0354	0.8292 ± 0.0680	0.6040 ± 0.1476	0.7630 ± 0.0717	**0.9836 ± 0.0101**

**TABLE 4:** Performance of five LDA inference methods under CV_ld_.

	Dataset	SDLDA	LDNFSGB	IPCARF	LDASR	LDA-SABC
Precision	LncRNADisease	0.8782 ± 0.0306	0.7782 ± 0.0270	0.7069 ± 0.0478	0.7695 ± 0.0393	**0.9052 ± 0.0241**
	MNDR	0.9178 ± 0.0154	0.8548 ± 0.0156	0.7693 ± 0.0850	0.8553 ± 0.0189	**0.9525 ± 0.0153**
Recall	LncRNADisease	0.7256 ± 0.0376	0.8169 ± 0.0408	0.6155 ± 0.0652	0.6836 ± 0.0342	**0.9074 ± 0.0329**
	MNDR	0.8824 ± 0.0198	0.8818 ± 0.0204	0.5034 ± 0.1469	0.8204 ± 0.0238	**0.9459 ± 0.0131**
Accuracy	LncRNADisease	0.8120 ± 0.0216	0.7916 ± 0.0256	0.6793 ± 0.0403	0.7385 ± 0.0283	**0.9058 ± 0.0183**
	MNDR	0.9015 ± 0.0114	0.8658 ± 0.0127	0.6793 ± 0.0753	0.8405 ± 0.0129	**0.9493 ± 0.0109**
F1 score	LncRNADisease	0.7939 ± 0.0260	0.7965 ± 0.0262	0.6563 ± 0.0492	0.7233 ± 0.0289	**0.9058 ± 0.0190**
	MNDR	0.8996 ± 0.0119	0.8679 ± 0.0129	0.5995 ± 0.1312	0.8371 ± 0.0137	**0.9491 ± 0.0108**
AUC	LncRNADisease	0.8774 ± 0.0200	0.8578 ± 0.0234	0.7384 ± 0.0466	0.8133 ± 0.0218	**0.9628 ± 0.0132**
	MNDR	0.9560 ± 0.0081	0.9346 ± 0.0074	0.7680 ± 0.0882	0.9143 ± 0.0112	**0.9878 ± 0.0046**
AUPR	LncRNADisease	0.8952 ± 0.0177	0.8489 ± 0.0289	0.7409 ± 0.0515	0.8131 ± 0.0277	**0.9606 ± 0.0150**
	MNDR	0.9639 ± 0.0063	0.9273 ± 0.0098	0.7689 ± 0.0924	0.9100 ± 0.0136	**0.9881 ± 0.0055**

*CV*_*l*_ was used to compare the performance of LDA-SABC with SDLDA, LDNFSGB, LDASR, and IPCAF when identifying diseases linking to a new lncRNA. Under *CV*_*l*_, all five methods randomly selected 80% of lncRNAs as the training set and used the remaining as the test set. The results are listed in Table 2 and Figure 2. We found that LDA-SABC outperformed in terms of precision, recall, accuracy, F1 score, AUC, and AUPR compared with the four classical LDA prediction algorithms. For example, LDA-SABC obtained the highest AUC values of 0.9328 and 0.9675, outperforming by 13.05% and 3.09% compared to those of the second best algorithm, on the LncRNADisease and MNDR databases, respectively. It also calculated the highest AUPR values of 0.9304 and 0.9703, outperforming by 8.43% and 1.76% compared to those of the second best algorithm, respectively. These results imply that LDA-SABC could accurately capture the underlying diseases linking to a new lncRNA.

*CV*_*d*_ was applied to compare the performance of LDA-SABC with SDLDA, LDNFSGB, LDASR, and IPCAF when identifying lncRNAs linking to a new disease. Under *CV*_*d*_, all five methods randomly selected 80% of diseases as the training set and used the remaining as the test set. As demonstrated in Table 3 and Figure 2, LDA-SABC significantly surpassed four other algorithms on the two datasets. For example, LDA-SABC obtained the highest AUC values of 0.9630 and 0.9860, outperforming by 8.42% and 3.01% compared to those of the second best algorithm (i.e., SDLDA), on the LncRNADisease and MNDR databases, respectively. It also calculated the highest AUPR values of 0.9605 and 0.9836, outperforming by 6.71% and 2.75% compared to those of the second best algorithm (i.e., SDLDA), on the LncRNADisease and MNDR databases, respectively. These results suggest that LDA-SABC could accurately infer potential lncRNAs linking to a new disease.

*CV*_*ld*_ is used to compare the performance of all five LDA inference methods when identifying new LDAs from unknown LDPs. Under *CV*_*ld*_, all five methods randomly selected 80% of LDPs as the training set and used the remaining as the test set. As demonstrated in Table 4 and Figure 2, LDA-SABC significantly improved LDA prediction in comparison with the four other methods. For example, LDA-SABC achieved the highest AUC values of 0.9628 and 0.9878, outperforming by 8.54% and 3.18% compared to those of the second best algorithm (i.e., SDLDA), on the LncRNADisease and MNDR databases, respectively. It also calculated the highest AUPR values of 0.9606 and 0.9881, outperforming by 6.54% and 2.42% compared to those of the second best algorithm (i.e., SDLDA), on the LncRNADisease and MNDR databases, respectively. Thus, LDA-SABC could more accurately infer the underlying LDAs through known LDAs.

### 3.3 Ablation study

**FIGURE 3:**
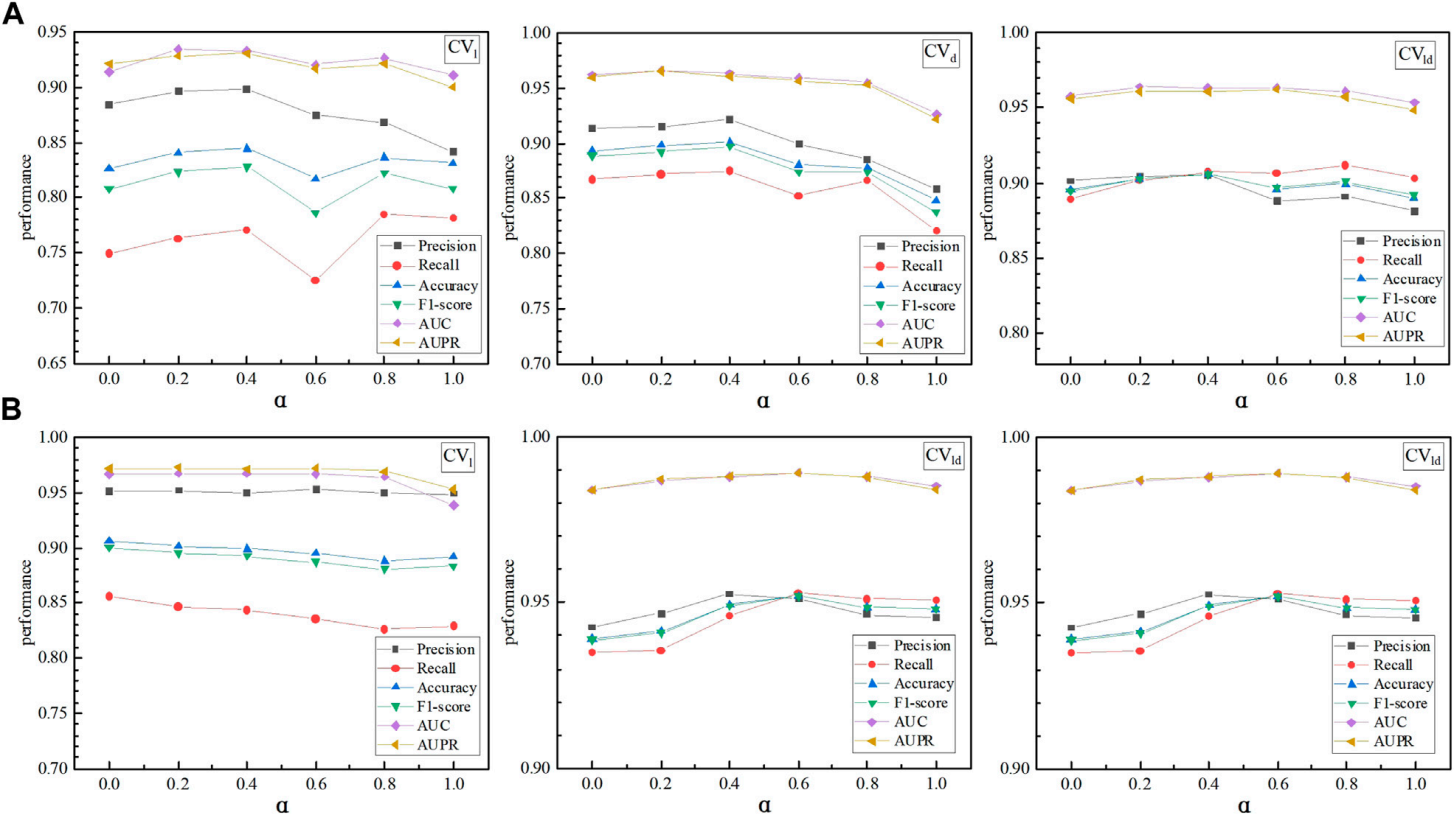
Effects of the parameters *α* and *β* on the LDA prediction performance: **(A)** performance of LDA-SABC based on *α* and *β* on LncRNADisease under *CV*_*l*_, *CV*_*d*_, and *CV*_*ld*_, respectively. **(B)** Performance of LDA-SABC based on different *α* and *β* values on MNDR under *CV*_*l*_, *CV*_*d*_, and *CV*_*ld*_, respectively.

LDA-SABC combined AdaBoost-CNN and LightGBM for LDA prediction. In model Ensemble, *α* and *β* were used to evaluate the effects of LDA-AdaBoost-CNN and LDA-LightGBM on the LDA inference performance, respectively. As shown in Figure 3, when *α* was set to 0, 0.2, 0.4, 0.6, 0.8, and 1, respectively, LDA-SABC achieved the best performance on the LncRNADisease and MNDR databases under fivefold CVs on lncRNAs, diseases, and LDPs. Supplementary Tables S1–S3 show the detailed performance of LDA-SABC when *α* was set to the above six values, respectively. Thus, we set *α* and *β* to 0.4 and 0.6, respectively.

To better understand the performance of ensemble learning, we compared LDA-SABC with other boosting algorithms, i.e., AdaBoost-CNN, AdaBoost, and LightGBM, under three different CVs. The boosting algorithms used the same feature extraction procedures as LDA-SABC except for using different boosting models for classifying unknown LDPs. Tables 5–7 show their LDA prediction performance under fivefold CVs on lncRNAs, diseases, and LDPs, respectively. The results demonstrate that LDA-SABC computed the best LDA inference accuracy on the two LDA databases under the three CVs in most cases, thereby elucidating the powerful LDP classification performance of our proposed ensemble learning model with LightGBM and AdaBoost-CNN.

**TABLE 5:** Performance of four boosting methods under CV_l_.

	Dataset	AdaBoost-CNN	AdaBoost	LightGBM	LDA-SABC
Precision	LncRNADisease	0.8412 ± 0.0584	0.7641 ± 0.0536	0.8836 ± 0.0354	**0.8980 ± 0.0306**
	MNDR	0.9486 ± 0.0217	0.8826 ± 0.0331	**0.9510 ± 0.0175**	0.9494 ± 0.0172
Recall	LncRNADisease	**0.7815 ± 0.0844**	0.7151 ± 0.0805	0.7494 ± 0.0765	0.7709 ± 0.0622
	MNDR	0.8295 ± 0.0728	0.8483 ± 0.0374	**0.8561 ± 0.0506**	0.8436 ± 0.0513
Accuracy	LncRNADisease	0.8307 ± 0.0309	0.7571 ± 0.0320	0.8261 ± 0.0523	**0.8444 ± 0.0445**
	MNDR	0.8916 ± 0.0419	0.8685 ± 0.0307	**0.9059 ± 0.0284**	0.8989 ± 0.0317
F1 score	LncRNADisease	0.8079 ± 0.0606	0.7359 ± 0.0525	0.8079 ± 0.0419	**0.8278 ± 0.0363**
	MNDR	0.8833 ± 0.0447	0.8644 ± 0.0265	**0.9002 ± 0.0293**	0.8925 ± 0.0307
AUC	LncRNADisease	0.9107 ± 0.0262	0.8252 ± 0.0308	0.9139 ± 0.0406	**0.9328 ± 0.0243**
	MNDR	0.9384 ± 0.0441	0.9314 ± 0.0216	0.9664 ± 0.0190	**0.9675 ± 0.0147**
AUPR	LncRNADisease	0.8997 ± 0.0575	0.8283 ± 0.0559	0.9209 ± 0.0262	**0.9304 ± 0.0252**
	MNDR	0.9526 ± 0.0250	0.9371 ± 0.0241	**0.9715 ± 0.0134**	0.9709 ± 0.0106

**TABLE 6:** Performance of four boosting methods under CV_d_.

	Dataset	AdaBoost-CNN	AdaBoost	LightGBM	LDA-SABC
Precision	LncRNADisease	0.8581 ± 0.0502	0.7788 ± 0.0560	0.9134 ± 0.0321	**0.9218 ± 0.0242**
	MNDR	0.9467 ± 0.0224	0.8750 ± 0.0380	0.9358 ± 0.0257	**0.9573 ± 0.0217**
Recall	LncRNADisease	0.8208 ± 0.0514	0.7746 ± 0.0576	0.8669 ± 0.0423	**0.8745 ± 0.0353**
	MNDR	0.9006 ± 0.0458	0.8521 ± 0.0665	0.9156 ± 0.0360	**0.9231 ± 0.0400**
Accuracy	LncRNADisease	0.8476 ± 0.0336	0.7832 ± 0.0288	0.8928 ± 0.0217	**0.9008 ± 0.0232**
	MNDR	0.9280 ± 0.0234	0.8769 ± 0.0177	0.9321 ± 0.0185	**0.9455 ± 0.0146**
F1 score	LncRNADisease	0.8376 ± 0.0378	0.7748 ± 0.0449	0.8884 ± 0.0218	**0.8970 ± 0.0218**
	MNDR	0.9223 ± 0.0260	0.8627 ± 0.0508	0.9254 ± 0.0288	**0.9394 ± 0.0260**
AUC	LncRNADisease	0.9263 ± 0.0226	0.8548 ± 0.0246	0.9615 ± 0.0124	**0.9630 ± 0.0122**
	MNDR	0.9758 ± 0.0107	0.9395 ± 0.0154	0.9825 ± 0.0068	**0.9860 ± 0.0057**
AUPR	LncRNADisease	0.9215 ± 0.0290	0.8453 ± 0.0581	0.9596 ± 0.0147	**0.9605 ± 0.0130**
	MNDR	0.9746 ± 0.0131	0.9290 ± 0.0367	0.9793 ± 0.0144	**0.9836 ± 0.0101**

**TABLE 7:** Performance of four boosting methods under CV_ld_.

	Dataset	AdaBoost-CNN	AdaBoost	LightGBM	LDA-SABC
Precision	LncRNADisease	0.8810 ± 0.0285	0.7989 ± 0.0262	0.9012 ± 0.0263	**0.9052 ± 0.0241**
	MNDR	0.9455 ± 0.0115	0.8755 ± 0.0157	0.9426 ± 0.0140	**0.9525 ± 0.0153**
Recall	LncRNADisease	0.9031 ± 0.0242	0.8040 ± 0.0323	0.8893 ± 0.0335	**0.9074 ± 0.0329**
	MNDR	**0.9507 ± 0.0119**	0.8691 ± 0.0230	0.9350 ± 0.0131	0.9459 ± 0.0131
Accuracy	LncRNADisease	0.8901 ± 0.0203	0.8003 ± 0.0214	0.8955 ± 0.0227	**0.9058 ± 0.0183**
	MNDR	0.9479 ± 0.0087	0.8726 ± 0.0129	0.9389 ± 0.0097	**0.9493 ± 0.0109**
F1 score	LncRNADisease	0.8916 ± 0.0194	0.8009 ± 0.0220	0.8948 ± 0.0232	**0.9058 ± 0.0190**
	MNDR	0.9480 ± 0.0087	0.8721 ± 0.0135	0.9387 ± 0.0096	**0.9491 ± 0.0108**
AUC	LncRNADisease	0.9532 ± 0.0144	0.8657 ± 0.0177	0.9575 ± 0.0122	**0.9628 ± 0.0132**
	MNDR	0.9850 ± 0.0043	0.9447 ± 0.0090	0.9839 ± 0.0042	**0.9878 ± 0.0046**
AUPR	LncRNADisease	0.9482 ± 0.0194	0.8610 ± 0.0189	0.9561 ± 0.0119	**0.9606 ± 0.0150**
	MNDR	0.9840 ± 0.0058	0.9454 ± 0.0106	0.9839 ± 0.0041	**0.9881 ± 0.0055**

### 3.4 Case study

Lung cancer is one of the most frequent malignant tumors and has a very high incidence and mortality rate. More importantly, its 5-year survival rate is much lower compared to other leading cancers (Huang et al., 2023). Non-small-cell lung cancer and lung adenocarcinoma (LUAD) are two prevalent lung cancers, wherein NSCLC accounts for approximately 85% of lung cancers (Tan et al., 2023) and LUAD is the most predominant subtype (Li et al., 2023). lncRNAs have close associations with various complex diseases and are potential biomarkers of many types of cancers. Therefore, it is very important to discover potential lncRNAs and further provide therapeutic options for lung cancer.

**FIGURE 4:**
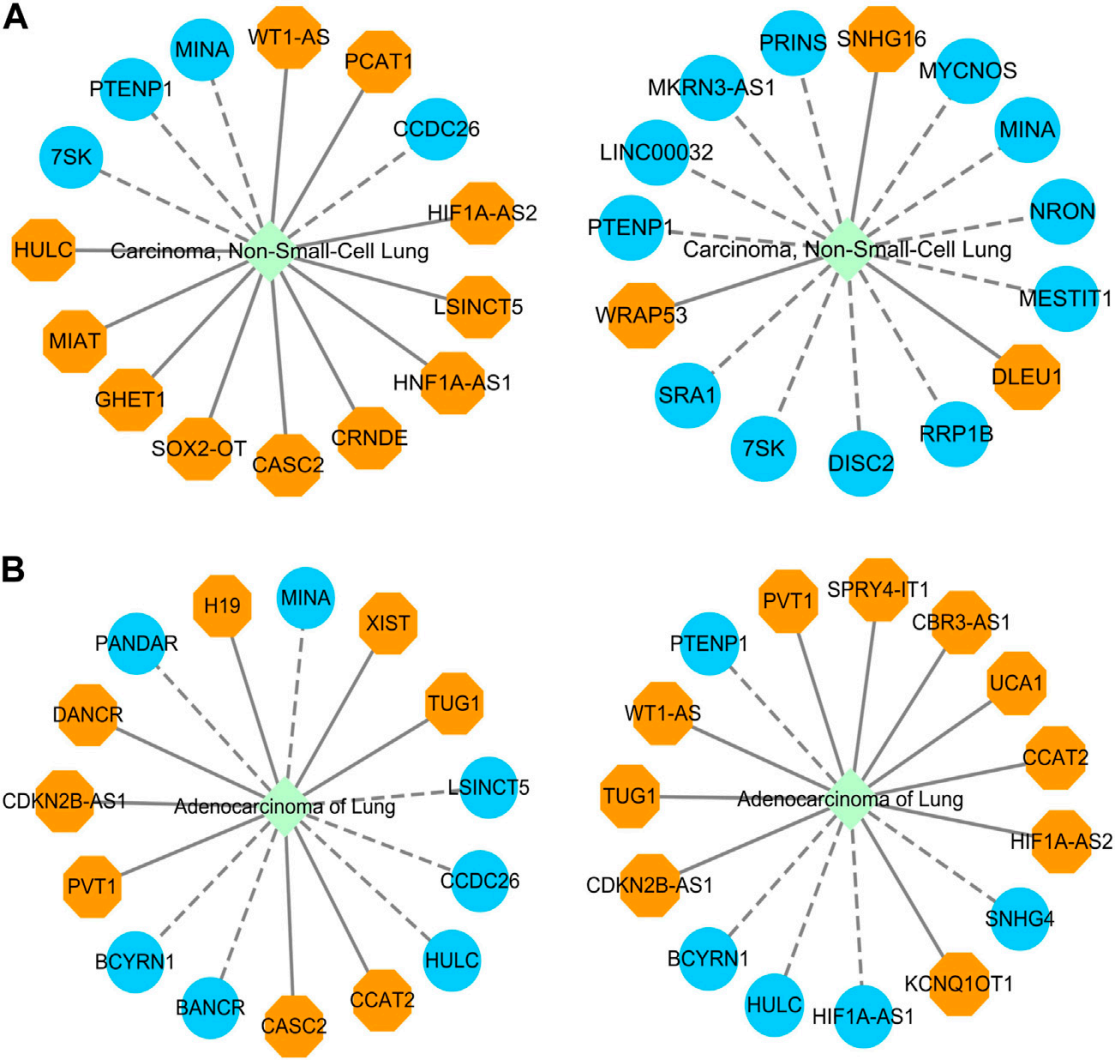
**(A)** Inferred top 15 lncRNAs associated with NSCLC on LncRNADisease and MNDR databases. **(B)** Inferred top 15 lncRNAs associated with LUAD on LncRNADisease and MNDR databases.

Through performance comparison, we validated the accurate LDA classification performance of LDA-SABC. Subsequently, we utilized LDA-SABC to discover the potential lncRNAs for NSCLC and LUAD. We computed the association probabilities between all lncRNAs and NSCLC and LUAD. Tables 8 and 9 demonstrate the top 15 lncRNAs with the highest association probability with NSCLC and LUAD among all lncRNAs which have no observed association with NSCLC and LUAD on the LncRNADisease and MNDR databases, respectively. Figure 4 elucidates two predicted LDA networks for NSCLC and LUAD.

**TABLE 8:** Predicted top 15 lncRNAs associated with NSCLC on LncRNADisease and MNDR.

LncRNADisease			MNDR		
Rank	LncRNA	Evidence	Rank	LncRNA	Evidence
1	HULC	Lnc2Cancer 3.0, RNADisease, and LncRNADisease v3.0	1	PTENP1	Unknown
2	MIAT	Lnc2Cancer 3.0, RNADisease, and LncRNADisease v3.0	2	WRAP53	RNADisease and Lnc2Cancer 3.0
3	MINA	Unknown	3	PRINS	Unknown
4	CCDC26	Unknown	4	MINA	Unknown
5	CRNDE	Lnc2Cancer 3.0, RNADisease, and LncRNADisease v3.0	5	RRP1B	Unknown
6	PCAT1	Lnc2Cancer 3.0, RNADisease, and LncRNADisease v3.0	6	MYCNOS	Unknown
7	HNF1A-AS1	Lnc2Cancer 3.0, RNADisease, and LncRNADisease v3.0	7	DLEU1	LncRNADisease v3.0
8	7SK	Unknown	8	LINC00032	Unknown
9	WT1-AS	LncRNADisease v3.0	9	SNHG16	Lnc2Cancer 3.0, RNADisease, and LncRNADisease v3.0
10	GHET1	RNADisease and LncRNADisease v3.0	10	SRA1	Unknown
11	SOX2-OT	RNADisease, and LncRNADisease v3.0	11	7SK	Unknown
12	PTENP1	Unknown	12	MKRN3-AS1	Unknown
13	CASC2	Lnc2Cancer 3.0, RNADisease, and LncRNADisease v3.0	13	DISC2	Unknown
14	HIF1A-AS2	LncRNADisease v3.0	14	NRON	Unknown
15	LSINCT5	Lnc2Cancer 3.0, RNADisease, and LncRNADisease v3.0	15	MESTIT1	Unknown

**TABLE 9:** Predicted top 15 lncRNAs associated with LUAD on LncRNADisease and MNDR.

LncRNADisease			MNDR		
Rank	LncRNA	Evidence	Rank	LncRNA	Evidence
1	CDKN2B-AS1	RNADisease and Lnc2Cancer 3.0	1	TUG1	RNADisease and LncRNADisease v3.0
2	PVT1	Lnc2Cancer 3.0, RNADisease, and LncRNADisease v3.0	2	CDKN2B-AS1	RNADisease and Lnc2Cancer 3.0
3	H19	Lnc2Cancer 3.0 and LncRNADisease v3.0	3	PVT1	Lnc2Cancer 3.0, RNADisease, and LncRNADisease v3.0
4	TUG1	RNADisease and LncRNADisease v3.0	4	UCA1	Lnc2Cancer 3.0, RNADisease, and LncRNADisease v3.0
5	CCAT2	Lnc2Cancer 3.0 and LncRNADisease v3.0	5	KCNQ1OT1	RNADisease and Lnc2Cancer 3.0
6	XIST	RNADisease and Lnc2Cancer 3.0	6	CBR3-AS1	LncRNADisease v3.0
7	HULC	Unknown	7	SNHG4	Unknown
8	DANCR	Lnc2Cancer 3.0, RNADisease, and LncRNADisease v3.0	8	WT1-AS	LncRNADisease v3.0
9	MINA	Unknown	9	SPRY4-IT1	RNADisease and Lnc2Cancer 3.0
10	BCYRN1	Unknown	10	BCYRN1	Unknown
11	BANCR	Unknown	11	HULC	Unknown
12	PANDAR	Unknown	12	PTENP1	Unknown
13	CASC2	Lnc2Cancer 3.0, RNADisease, and LncRNADisease v3.0	13	HIF1A-AS1	RNADisease
14	LSINCT5	Unknown	14	CCAT2	Lnc2Cancer 3.0, and LncRNADisease v3.0
15	CCDC26	Unknown	15	HIF1A-AS2	LncRNADisease v3.0

Among the inferred top 15 lncRNAs associated with NSCLC, 11 and 3 lncRNAs, predicted on the LncRNADisease and MNDR databases, have been confirmed by Lnc2Cancer 3.0 (Gao et al., 2021), LncRNADisease v3.0 (Lin et al., 2023b), and/or RNADisease (Chen et al., 2023), respectively. Particularly, 7SK was linked to NSCLC, which was ranked 8 and 11, respectively. lncRNA 7SK acts as a transcription regulator. Its exosomal delivery could inhibit the proliferation and aggressiveness of tumor cells in triple-negative breast cancer (Farhadi et al., 2023). Furthermore, 7SK could suppress human tongue squamous carcinoma (Zhang et al., 2021). 7SK was predicted to be associated with NSCLC, which needs further confirmation.

Among the inferred top 15 lncRNAs associated with LUAD, 8 and 11 lncRNAs, predicted on the LncRNADisease and MNDR databases, have been reported by Lnc2Cancer 3.0, LncRNADisease v3.0, and/or RNADisease, respectively. We found that HULC could be associated with LUAD, which was ranked 7 and 11, respectively. HULC is an oncogenic lncRNA and may serve as a prognostic biomarker of hepatocellular carcinoma development (Liu S. et al., 2023). Moreover, it displays the potential to be a novel biomarker for assisting acute myocardial infarction diagnosis when combined with other biomarkers (Xie et al., 2022).

## 4 Discussion and conclusion

Inferring possible LDAs can advance our understanding of human complex diseases in the context of lncRNAs. However, traditional experimental techniques for LDA prediction are costly, laborious, and time-consuming, which restricts the number of the verified LDAs. Thus, substantive computational frameworks have been exploited. In this manuscript, we proposed a novel computational LDA inference framework LDA-SABC by combining SVD and an ensemble model of LightGBM and AdaBoost-CNN.

LDA-SABC first acquired LDP linear features using SVD. Next, it computed the association probability for each LDP with LDA-LightGBM and LDA-AdaBoost-CNN. Finally, all LDPs were classified through ensemble learning. To illustrate the effectiveness of LDA-SABC, it was compared with four classical computational methods (SDLDA, LDNFSGB, IPCARF, and LDASR) under three CVs. The results elucidated that its performance was significantly improved. To validate the performance of LDA-SABC, we further performed case studies to find potential biomarkers of NSCLC and LUAD and discovered the top 15 lncRNAs linked to them from all unknown LDPs. The results demonstrated that among the inferred top lncRNAs reported by RNADisease, LncRNADisease v3.0, or/and Lnc2Cancer 3.0 databases, 7SK and HULC could have a relationship with NSCLC and LUAD, respectively.

The novelty of this study is the use of SVD for extracting LDP features and designing an ensemble model with LightGBM and AdaBoost-CNN for improving the LDA prediction accuracy. Differing from traditional LDA prediction performance validation, LDA-SABC was assessed under fivefold CVs on lncRNAs, diseases, and LDPs. However, in the process of negative LDA selection, a random selection strategy was adopted, which affected the overall performance of the model. In the future, we will design a reasonable negative LDA selection strategy based on positive-unlabeled learning. More importantly, we will still explore a stronger classification model for LDP classification by integrating various data and deep learning methods. We hope that our proposed LDA-SABC could contribute to the lncRNA biomarker discovery of various complex diseases, especially cancers, and further help find new therapeutic options for various types of cancers.

## Data Availability

Publicly available datasets were analyzed in this study. These data can be found at: https://github.com/plhhnu/LDA-SABC.
