# Supplementary material for: Finding potential lncRNA–disease associations using a boosting-based ensemble learning model
Source: Front Genet. 2024 Mar 1;15:1356205. doi: 10.3389/fgene.2024.1356205 (PMC10940470; doi:10.3389/fgene.2024.1356205)
Supplement: Supplementary file 1 [file Table1.PDF]

Supplementary Materials

1 TABLES

Table S1. The effect different α and β values on the LDA prediction performance under CV_l

	Dataset	$\alpha = 0$	$\alpha = 0.2$	$\alpha = 0.4$	$\alpha = 0.6$	$\alpha = 0.8$	$\alpha = 1$
Precision	lncRNADisease	0.8836±0.0354	0.8957±0.0337	**0.8980 ± 0.0306**	0.8747±0.0632	0.8678±0.0443	0.8412±0.0584
	MNDR	0.9510±0.0175	0.9518±0.0148	0.9494±0.0172	**0.9522 ± 0.0190**	0.9490±0.0208	0.9486±0.0217
Recall	lncRNADisease	0.7494±0.0765	0.7632±0.0532	0.7709±0.0622	0.7249±0.1151	**0.7845 ± 0.0736**	0.7815±0.0844
	MNDR	**0.8561 ± 0.0506**	0.8463±0.0457	0.8436±0.0513	0.8356±0.0885	0.8262±0.0877	0.8295±0.0728
Accuracy	lncRNADisease	0.8928±0.0217	0.8405±0.0371	**0.8444 ± 0.0446**	0.8167±0.0663	0.8362±0.0400	0.8307±0.0309
	MNDR	**0.9059 ± 0.0284**	0.9012±0.0272	0.8989±0.0317	0.8949±0.0475	0.8881±0.0499	0.8916±0.0419
F1-score	lncRNADisease	0.8079±0.0419	0.8230±0.0355	**0.8278 ± 0.0363**	0.7863±0.0833	0.8219±0.0477	0.8079±0.0606
	MNDR	**0.9002 ± 0.0293**	0.8952±0.0252	0.8925±0.0307	0.8868±0.0513	0.8804±0.0502	0.8833±0.0447
AUC	lncRNADisease	0.9139±0.0406	**0.9340 ± 0.0212**	0.9328±0.0243	0.9203±0.0344	0.9259±0.0245	0.9107±0.0262
	MNDR	0.9664±0.0190	0.9675±0.0162	**0.9675 ± 0.0147**	0.9671±0.0209	0.9641±0.0202	0.9384±0.0441
AUPR	lncRNADisease	0.9209±0.0262	0.9281±0.0277	**0.9304 ± 0.0252**	0.9169±0.0405	0.9209±0.0287	0.8997±0.0575
	MNDR	0.9715±0.0134	**0.9723 ± 0.0091**	0.9709±0.0106	0.9721±0.0132	0.9697±0.0128	0.9526±0.0250

Table S2. The effect different α and β values on the LDA prediction performance under CV_d

	Dataset	$\alpha = 0$	$\alpha = 0.2$	$\alpha = 0.4$	$\alpha = 0.6$	$\alpha = 0.8$	$\alpha = 1$
Precision	lncRNADisease	0.9134±0.0321	0.9150±0.0280	**0.9218 ± 0.0242**	0.8986±0.0243	0.8853±0.0207	0.8581±0.0502
	MNDR	0.9358±0.0257	0.9511±0.0164	0.9573±0.0217	**0.9590 ± 0.0227**	0.9486±0.0213	0.9467±0.0224
Recall	lncRNADisease	0.8669±0.0423	0.8716±0.0454	**0.8745 ± 0.0353**	0.8524±0.0651	0.8660±0.0438	0.8208±0.0514
	MNDR	0.9156±0.0360	**0.9243 ± 0.0275**	0.9231±0.0400	0.8693±0.0950	0.8745±0.0929	0.9006±0.0458
Accuracy	lncRNADisease	0.8928±0.0217	0.8978±0.0180	**0.9008 ± 0.0232**	0.8800±0.0332	0.8776±0.0195	0.8476±0.0336
	MNDR	0.9321±0.0185	0.9409±0.0147	**0.9445 ± 0.0146**	0.9225±0.0464	0.9171±0.0446	0.9280±0.0234
F1-score	lncRNADisease	0.8884±0.0218	0.8918±0.0123	**0.8970 ± 0.0218**	0.8737±0.0422	0.8736±0.0244	0.8376±0.0378
	MNDR	0.9254±0.0288	0.9373±0.0184	**0.9394 ± 0.0260**	0.9093±0.0615	0.9074±0.0591	0.9223±0.0260
AUC	lncRNADisease	0.9615±0.0124	**0.9658 ± 0.0053**	0.9630±0.0122	0.9590±0.0151	0.9549±0.0091	0.9263±0.0226
	MNDR	0.9825±0.0068	0.9855±0.0054	**0.9860 ± 0.0057**	0.9833±0.0079	0.9822±0.0086	0.9758±0.0107
AUPR	lncRNADisease	0.9596±0.0147	**0.9650 ± 0.0111**	0.9605±0.0130	0.9563±0.0165	0.9528±0.0155	0.9215±0.0290
	MNDR	0.9793±0.0144	**0.9844 ± 0.0072**	0.9836±0.0101	0.9801±0.0159	0.9792±0.0156	0.9746±0.0131

Table S3. The effect different α and β values on the LDA prediction performance under CV_{ld}

	Dataset	$\alpha = 0$	$\alpha = 0.2$	$\alpha = 0.4$	$\alpha = 0.6$	$\alpha = 0.8$	$\alpha = 1$
Precision	lncRNADisease	0.9012±0.0263	0.9046±0.0244	$\mathbf{0.9052 \pm 0.0241}$	0.8883±0.0230	0.8910±0.0311	0.8810±0.0285
	MNDR	0.9426±0.0140	0.9466±0.0144	$\mathbf{0.9525 \pm 0.0153}$	0.9511±0.0090	0.9463±0.0140	0.9455±0.0115
Recall	lncRNADisease	0.8893±0.0335	0.9015±0.0224	0.9074±0.0329	0.9064±0.0263	$\mathbf{0.9117 \pm 0.0299}$	0.9031±0.0242
	MNDR	0.9350±0.0131	0.9356±0.0143	0.9459±0.0131	$\mathbf{0.9529 \pm 0.0140}$	0.9510±0.0131	0.9507±0.0119
Accuracy	lncRNADisease	0.8955±0.0227	0.9030±0.0192	$\mathbf{0.9058 \pm 0.0183}$	0.8959±0.0081	0.8995±0.0229	0.8901±0.0203
	MNDR	0.9389±0.0097	0.9413±0.0077	0.9493±0.0109	$\mathbf{0.9519 \pm 0.0081}$	0.9484±0.0089	0.9479±0.0087
F1-score	lncRNADisease	0.8948±0.0232	0.9029±0.0191	$\mathbf{0.9058 \pm 0.0190}$	0.8969±0.0172	0.9008±0.0224	0.8916±0.0194
	MNDR	0.9387±0.0096	0.9409±0.0077	0.9491±0.0108	$\mathbf{0.9519 \pm 0.0082}$	0.9485±0.0088	0.9480±0.0087
AUC	lncRNADisease	0.9575±0.0122	$\mathbf{0.9637 \pm 0.0137}$	0.9628±0.0132	0.9634±0.0120	0.9609±0.0147	0.9532±0.0144
	MNDR	0.9839±0.0042	0.9866±0.0027	0.9878±0.0046	$\mathbf{0.9891 \pm 0.0030}$	0.9880±0.0035	0.9850±0.0043
AUPR	lncRNADisease	0.9561±0.0119	$\mathbf{0.9607 \pm 0.0193}$	0.9606±0.0150	0.9621±0.0133	0.9571±0.0189	0.9482±0.0194
	MNDR	0.9839±0.0041	0.9872±0.0029	0.9881±0.0055	$\mathbf{0.9891 \pm 0.0031}$	0.9878±0.0042	0.9840±0.0058
